# The Effects of Insole-Based Visual Feedback on Weight-Bearing in Patients Undergoing Total Hip Replacement

**DOI:** 10.3390/ijerph18073346

**Published:** 2021-03-24

**Authors:** Luca Marin, Matteo Vandoni, Giancarlo Zaza, Massimiliano Febbi, Luisella Pedrotti, Matteo Chiodaroli, Nicola Lovecchio, Federica Manzoni

**Affiliations:** 1 Department of Rehabilitation, Faculty of Medicine, University of Ostrava, 70103 Ostrava, Czech Republic; luca.marin@unipv.it (L.M.); massimilianofebbi@gmail.com (M.F.); 2 Laboratory for Rehabilitation, Medicine and Sport (LARMS), 00100 Rome, Italy; giancarlo.zaza@me.com; 3 Department of Rehabilitation, Città di Pavia Hospital, 27100 Pavia, Italy; kiodabagei@gmail.com; 4 Laboratory of Adapted Motor Activity (LAMA), Department of Public Health, Experimental and Forensic Medicine, University of Pavia, 27100 Pavia, Italy; nicola.lovecchio@unipv.it; 5 Department of Pediatric Diagnostic Surgical Clinical Science, Section of Pathologies of the Musculoskeleta System, Orthopedics Unit, University of Pavia, 27100 Pavia, Italy; luisella.pedrotti@unipv.it; 6 Unit of Biostatistics and Clinical Epidemiology, Department of Public Health, Experimental and Forensic Medicine, University of Pavia, 27100 Pavia, Italy; federica.manzoni@unipv.it; 7 Health Promotion—Environmental Epidemiology Unit, Hygiene and Health Prevention Department, Health Protection Agency, 27100 Pavia, Italy

**Keywords:** sensorized insoles, hip replacement, visual feedback, rehabilitation, weight bearing, clinical measure

## Abstract

This study aimed to investigate the visual biofeedback effect of a sensorized system for plantar pressure dynamic evaluation of in patients with a total hip replacement. Experimental group followed the rehabilitation training wearing sensorized insoles that provided images on three monitors. The control group followed the verbal instructions of physiotherapists during training. Weight bearing percentage healthy limb (WBPH), weight bearing percentage surgical limb (WBPS), swing healthy limb (SWH) and swing surgical limb (SWS) improved significantly more in the experimental group. The results underline the effectiveness of visual biofeedback based on sensorized system with dynamic evaluation of the plantar pressure.

## 1. Introduction

Osteoarthritis (OA) is a degenerative disease characterized by loss of articular cartilage, formation of osteophytes, synovitis and then weakening of the periarticular muscles. The OA primary symptoms include articular pain and manifest limitation on range of motion (ROM). Its progression is usually slow but it lead to disability depending on the site of affection: most frequently hands, articular vertebrae facets, hip and knee [1]. In particular, people affected by hip osteoarthritis develop adaptation to compensate the decreased ROM that limit one of the most important daily living activity: walking [2]. The total hip replacement (THR), become a resolutive procedure generally adopted for those patients who reach a level of impairment not treatable by means of other types of therapy [3]. Nevertheless, even after surgery, gait cycle compensations may persist over time [2] and can also be observed at 6- and 12-months follow up [4]. For this reason, the correct weight-bearing distribution on both lower limbs during walking is one of the priorities during the first phases of a rehabilitation program. In point of this, patients are often controlled and guided by physical therapist (PT) through verbal instructions, even if exercises supported by biofeedback (BF) systems are also used [5]. In the immediate post-surgery period, the use of a BF enhance the recovery of the correct weight-bearing distribution on both limbs during walking [2,6]. Often, in a clinical context, the physiotherapist becomes the irreplaceable guide for the patient generating a sort of dependance while the aim of the treatment should be to promote patient’s independence, self-control and proprioceptive capacities. For these reasons, the use of safe and feasible methods that could help patients in self-regulation may be seen as a new strong approach for the patients’ care.

Currently there are several wearable devices to evaluate the general load and feet pressure [7]. The most reliable tools for static and dynamic analysis of pressure are the platform of force [8,9]. Although this system has been widely used in several research fields and for clinical evaluation (i.e., cerebral palsy sequelae), it is very expensive and hard to adopt in everyday clinical practice, since it is dependent on the laboratory space and on specialized operators.

In order to analyze human motion of the weakest populations [10] and in specific contexts, wearable systems, using inertial motion sensors and pressure sensors, have been recently created [11,12,13,14].

Therefore, this research aims to study the effects of the use of a sensorized system for the dynamic assessment of plantar pressure as a surrogate of verbal PT suggestions. The primary aim of this study is to compare the difference in weight bearing distribution on the lower limbs after THR between the experimental group (EG) who followed a THR rehabilitation protocol by using a visual BF and the control group (CG) who followed a protocol based on verbal instructions given by the PT (without the visual BF).

## 2. Materials and Methods

### 2.1. Trial Design

The present study is an open randomized controlled trial, with parallel groups, in which only the PT evaluator was blinded. The study protocol was approved by the local ethical board (‘Area Vasta Pavia’ Ethical Committee Prot.20180036031) and carried out in accordance with the CONSORT 2010 Statement Guidelines [15] as reported in Supplementary section Consort Checklist (S1) and with the current version of the World Medical Association Declaration of Helsinki [16].

The study was registered in clinicaltrial.gov (ClinicalTrials.gov Identifier NCT04268082) after the recruitment of patients. The authors confirm that the current and related trials for this intervention are registered.

### 2.2. Participants: Eligibility Criteria and Sample Size Consideration

Inclusion criteria were: 75 ≤ years of age, both genders, absence of conditions that could reduce the neural afference or alter visual ability, heterometry of lower limbs ≤ 0.5 cm, Mini-Mental State Examination (MMSE) score ≥ 24. Exclusion criteria consisted in a previous hip replacement in the contralateral side, knee prosthesis or other skeletal injuries (arthrosis, past meniscal injuries and ankle orthosis) and reported anamnesis of pathologies that could led to neurological impairment (i.e., diabetes or vestibular corruptions). Since in literature no univocal indications for an effect size calculation can be found up to date, a proportion of individuals with correct weight bearing (WB) was hypothesized. In order to detect a 90% proportion of subjects with a correct WB in the experimental group compared to a 50% proportion in the control group, 19 patients per group ensured an 81% power, with a 0.05 alpha significance level. Considering a 5% drop out rate, it was necessary to enroll 20 patients per group.

### 2.3. Sensorized Insoles

**Figure 1:**
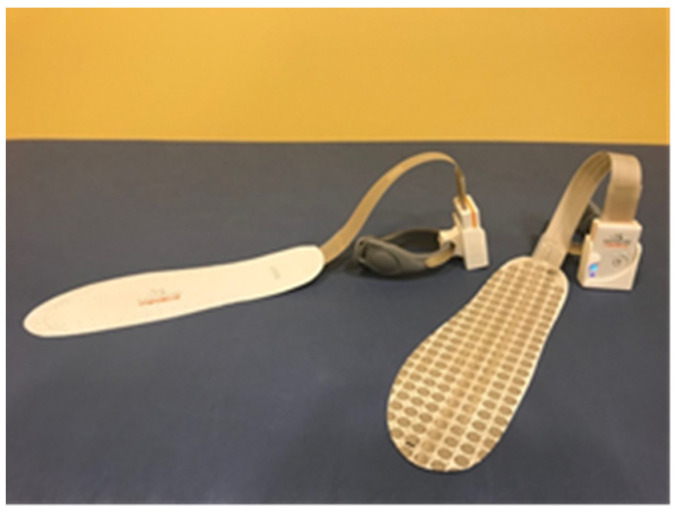
Sensorized insoles.

A couple of sensorized insoles (FlexInFit ^®^, Sensormedica, Guidonia Montecelio, Rome, Italy) were used inside the shoes since the reduced thickness (0.3 mm; Figure 1) made them a wearable tool (Figure 1).

The insoles use wireless Bluetooth technology to broadcast the signals to an external processing unit guaranteeing free movement and no-pattern modification. These insoles have 420 sensors, that allow a frequent data flow (from 25 to 50 Hz) about the feet contact on the ground and then a real-time representation of the footprint on the screen: used as BF for weight-bearing distribution.

### 2.4. Interventions

Two days after surgery (DAS) the eligible patients were randomized with the simple method with an online generator [17] with a blind control to guarantee a balanced distribution of male and female and the side of intervention.. Those who confirmed their free participation were asked to sign an informed consent form. Participants were guaranteed to interrupt at any time their adherence to the protocol according to their free choice or in case of complications that could affect the rehabilitation program.

**Figure 2:**
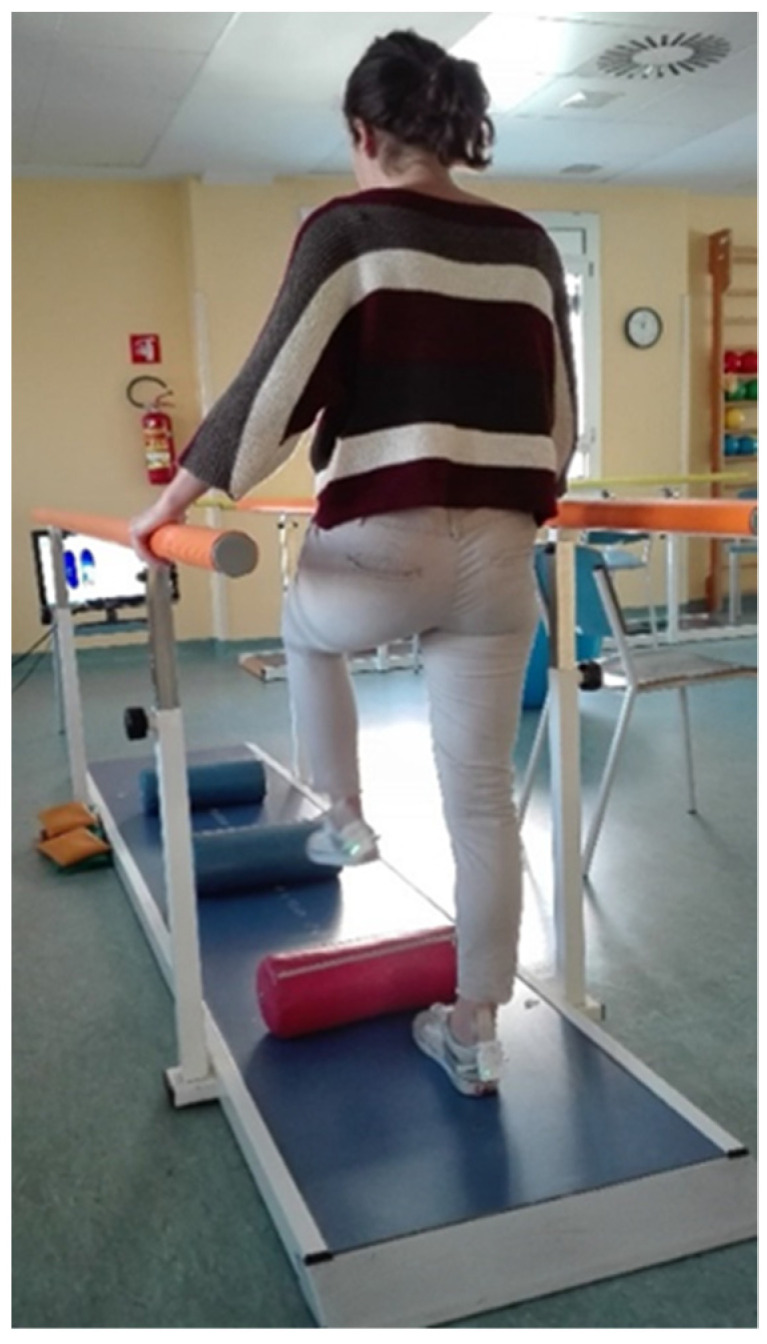
Experimental setting.

During the second DAS all patients (*n*= 40) were assigned to the EG (*n* = 19; M = 10; 10 right side under surgery) or CG (*n*= 21; M = 10; 10 right side under surgery;) and followed two distinct methodological approach (timeline signed in Table 1) to the same rehabilitation intervention (PF-Fisiot.02), in the same clinical setting (Figure 2). All patients were right foot dominant.

**Figure 3:**
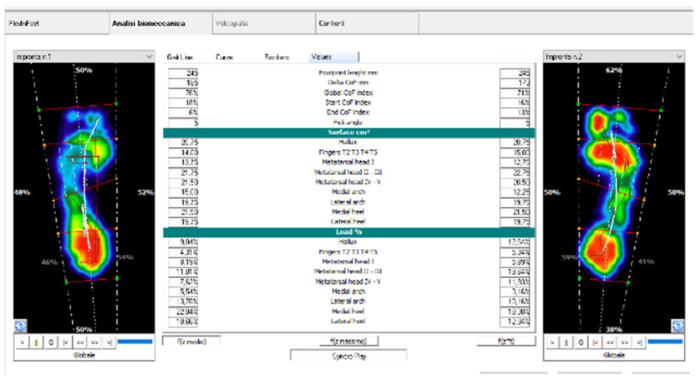
Software workflow.

**Figure 4:**
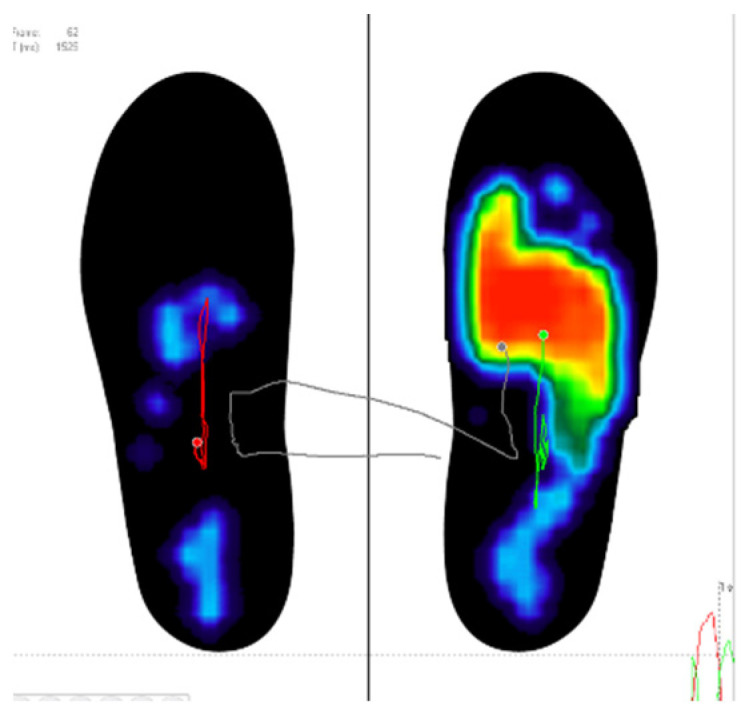
Visual Biofeedback.

The EG patients self-adjusted their gait patterns by wearing the FlexInFit^®^ sensorized insoles and using the FreeStep software’s graphical interface as a visual BF. In particular, two screens at the end of the parallel bars were placed (60 cm. height from the ground) to permit the BF observing the output representation of the feet pressure (Figure 3 and Figure 4). In particular, patients were requested to get two plantar images of the same color and form keeping the center of pressure at the center of the two limbs. A third screen was placed on the wall (130 cm from the ground) and used during no-assisted gait or during gait with crutches.

The CG, instead, were supported by the same PT with verbal feedback to focus the attention on the symmetrical distribution of the body weight and on the correct gait patterns. The rehabilitation intervention was carried out during 10 sessions in the clinical gym. The clinical purpose was the earlier rehabilitation for the independence during walking with a particular focus on the equal distribution of the BW on the lower limbs.

Walking within the parallel bars consisted in three different performances in the paths. The first consisted in a free walk using the bar as support to regain the alternance of gait stride. The second set of trials was a walking on seven proprioceptive pads while the third set consisted in performing high steps to pass over three cylindrical tubes in order to markedly flex the knee and to improve the clearance of the feet.

Participants had to walk along the paths for eight times, five for each set.

In advance, they walked outside the parallel bars using crunches and, when they improved sufficiently, without any support. At the beginning and at the end of each walk, EG participants used the images on the monitors to distribute the BW on both limbs. Patients tried to get on the screen two plantar images of the same color and form. They were asked to focus their attention on the representation of the footprints. They also tried to keep the center of pressure at the middle of the two images of the feet. The aims, indeed, was a self-corrective action by each patient to magnify the plantar perception during loading response.

The sequence of trials was identical for the two groups and covered a period of 45 min while a self-chosen time of rest was guaranteed to every patient between the repetition. For both groups, the intervention started on 4 DAS and ended on 10 DAS. Patients who interrupted the rehabilitation program for more than one day were excluded from the study.

### 2.5. Measures

At baseline (4 DAS) and at the end of the rehabilitation program (10 DAS) a series of measures were administered in order to evaluate the distribution of the body weight on the lower limbs, the general ability to walk and the level of pain.

A stabilometric platform (Dynamic, Sensormedica, Guidonia Montecelio, Rome, Italy) was used to define the body weight distribution, the Six Minutes Walking Test (6MWT) was performed for the ability to walk. The 6MWT is a practical validated simple test that requires a 20 m hallway in which patients walked continuously on a flat, hard surface in a period of 6 min [18]. At the end of the test the total distance (m) walked by patients was recorded.

Finally the Numeric Rating Scale (NRS) as index of pain [19,20,21].

In particular, the assessment on the platform consists in collecting multiple outcomes as define below:

- Weight Bearing Absolute Healthy limb (WBAH; Kg)
- Weight Bearing Absolute Surgical limb (WBAS; Kg)
- Weight Bearing Percentage Healthy limb (WBPH; %)
- Weight Bearing Percentage Surgical limb (WBPS; %)
- Step Length Healthy limb (SLH; cm)
- Step Length Surgical limb (SLS; cm)
- Swing Healthy limb (SHL; ms)
- Swing Surgical limb (SSL; ms)
- Double Support Time (DST; ms)

### 2.6. Blinding

Patients and PT in the clinical setting during the rehabilitation program were not blinded while the evaluator who collected the measures at baseline and the end of the protocol was blinded to the treatment allocation.

### 2.7. Statistical Methods

Quantitative variables were described as mean and standard deviation, if normally distributed (Shapiro-Wilk test), as median and interquartile range if not normally distributed; qualitative variables were expressed with counts and percentages. Associations between qualitative variables were studied with the Pearson’s chi-squared test. Univariate comparisons between two groups at baseline were made either with Student’s *t*-test or with the analogous nonparametric Mann-Whitney U test for quantitative variables depending on the normality of the distribution.

Univariate and multivariate linear regression models for repeated data over time, with interaction between time and treatment, were used to compare the measurements between the two groups at different time points. Univariate and multivariate linear regression models for repeated data over time were performed within each group to compare the measurements under study at different time assessments. Opportune adjustments for WBAH and WBAS at baseline were provided in the regression models for respectively WBAH and WBAS variation in time between the two groups, since a statistically significant difference in WBAH and WBAS values at baseline between the two groups was observed. Appropriate logarithmic transformations of the outcome variables in the regression models were provided, if necessary, after checking for the normality of the distribution. All the tests were two-sided. The significance level was set at alpha 0.05. Data analysis were performed with the STATA statistical software version 14 (Stata Corporation, College Station, TX, USA, 2015).

## 3. Results

In general, the patients were 169 cm (9.6) height and 78 kg (10.4) weight. The two groups differed for weight (Table 2) and also for BMI. During the hospitalization all people followed a controlled diet that led to a general weight loss of 2 kg (0.85).

WBAH decreased significatively in both group such as the WBAS increased (Table 3). The absolute differences between the two side (ΔWBA) decreased with an important matching in EC (two kg of difference). In particular, these mean differences revealed significative differences between the two group (*p* < 0.05).

We can empirically observe that the sum of the weight on the two side overreached the personal weight with minimal differences between groups (Table 4).

**Figure 5:**
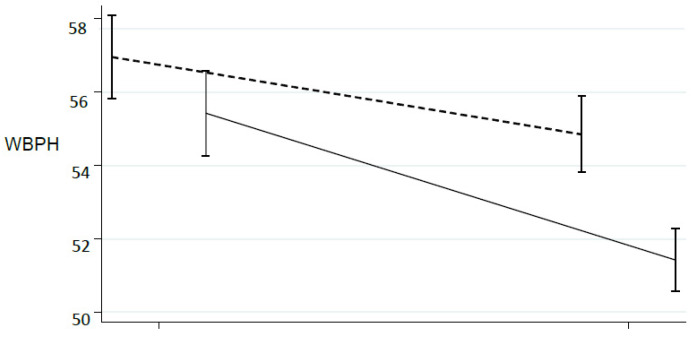
Percentage of WB during rehabilitation program about the healthy limb. CG: dot line; EG: solid line.

**Figure 6:**
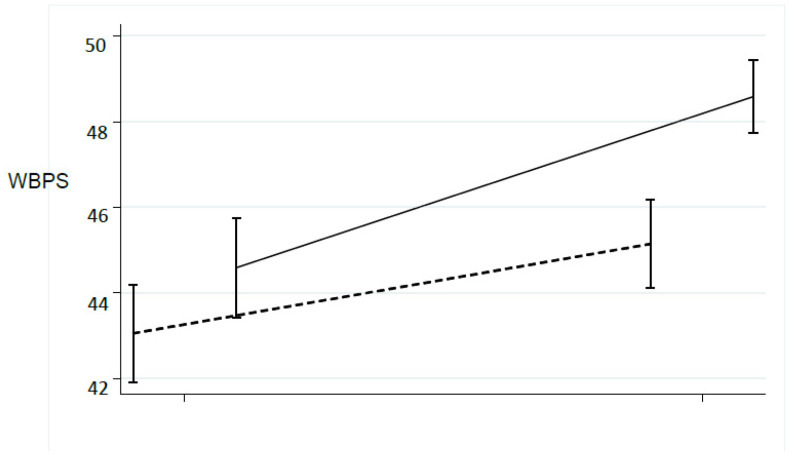
Percentage of WB during rehabilitation program about the surgery limb. CG: dot line; EG: solid line.

This trend could be emphasized scaling the absolute weight: the Figure 2 and Figure 3, showed the trend according to the percentage of WB (WBPH, WBPS): are evident that the two side approaches near 50% (Figure 5 and Figure 6).

Considering the step length (SLH, SLS) both sides reached similar results during the post-rehabilitation assessment. Within the EG the SLH and SLS become 38.2 cm (5.9) and 39.3 cm (6.9) while the CG showing substantially equal results between side of 36.3 cm (5.4).

The two groups significantly reduced the duration of the steps according to DST and swing of the limbs (data not showed). On average the EG used a total of 1772 ms to complete the gait (sum of DST, SHL and SSL) while CG a very similar period (1776 ms).

Also, the 6MWT (Table 4) revealed a significative increasement withing group with a total of covered meters in post analysis at most similar (close to 309 m). The self-perception of pain (NRS) decreased along the rehabilitation program: significant differences in pre-post comparison within group. No differences between group at 10 DAS (Table 3).

## 4. Discussion

Rehabilitation after surgery is a crucial phase in patients’ recovery and health status. Several studies highlighted the efficacy of a functional approach to maximize the effects of the administered treatment [6,22]. A study by Ewen et al. [2] demonstrated that after THR surgery some WB alterations persisted, which lead to gait alterations as a compensation to avoid pain and to keep the stability. Therefore, an early rehabilitation of the pair WB become crucial as reported in recent research [23,24,25]. The PT verbal suggestions are the most frequently used technique to regain the correct WB even if they become more effective when combined with BF [5]. The BF systems used in some studies require hardware that modify the free human movements leading to modified patterns while BF wireless system based on in-shoe pressure sensors were feasible for normal motions.

Our results highlighted significative matching between the WB on the two limbs in both groups with a greater improvement observed in EG. These outcomes are consistent with other studies that have stressed the effectiveness of BF systems on the correct WB distribution on the lower limbs [1,26,27] even if in this case a shorter duration of the intervention was adopted. The analysis of the WB percentage was interesting: statistically significant differences were found indicating a trend to report the 50% of the weight on each side. The EC better approached the 50% of the WB (Figure 5 and Figure 6).

We therefore believe that it is crucial to recover soon after surgery the balance of the load on the single limb, especially on the operated one, in order to prevent incorrect patterns that may lead to joint overloads, as was previously reported in literature [11].

In general, the gait parameters (SL, SW, DST) after the two intervention were comparable confirming the validity of traditional PT intervention [22] such as the perception of pain and the autonomy (6MWT). Essentially, our results showed a better pattern to manage the foot pressure by the EG by the use of BF [5].

The authors, during clinical practice focused the attention to the ability to symmetrically distribute the weight on both limbs EMG analysis [2] showed ‘no-normal’ patterns of muscle activation in the patients after THR. These anomalies did not only affect the muscles damaged by surgery, such as the gluteus medius, but also others leg muscles, leading to alterations in gait pattern.

According to Schacks’ [28] theory of the cognitive architecture of complex movements, the outcomes might be also due to a modification of the sensor motor control, that is the regulation of functional units by using afferent feedback, effectors and perceptual effect representations. The perceptual effect representation incorporates action-specific information (e.g., spatial-temporal adjustments). Improvement in kinematic outcomes may be the result of the adapted perceptual effect representation and probably derives from a change of basic action concept structure (BACs) (mental representation) [29,30,31,32]. In this sense the visual BF may have helped the EG ameliorate the BACs [33]. By the BF, the EG participants carried out a self-corrective gait pattern probably with a more proprioceptive attention on plantar foot pressure.

## 5. Conclusions

Our results highlight a substantial improvement in the weight bearing distribution for the EG compared to the CG. This study supports the hypothesis that a visual biofeedback, based on the use of sensorized insoles, can act as a support for the rehabilitation treatment in order to regain the correct weight-bearing. This process may facilitate the performance of the rehabilitation activities magnifying the peripherical nervous system caption.

## Figures and Tables

**Table 1:** Protocol timeline.

	Days after Surgery								
	2	3	4	5	6	7	8	9	10
Enrollment	x								
Randomization		x							
Evaluation			x						x
Intervention			x	x	x	x	x	x	x

**Table 2:** Demographic description of the groups.

Outcomes	*All*	*EG (n = 19)*	*CG (n = 21)*	*p Value*
**Age (years)**	62.58 (9.06)	64.12 (7.48)	61.30 (9.74)	0.058
**Height (cm)**	169.35 (9.99)	166.42 (9.77)	172.00 (9.65)	0.077
**Weight (kg)**	79.85 (12.94)	72.74 (9.49)	86.29 (12.41)	**<0.001**
**BMI**	27.87 (4.09)	26.34 (3.6)	29.25 (4.1)	**0.023**

Data are reported as mean and (standard deviation).

**Table 3:** Outcomes differences between groups and in the time.

Outcomes	EG		CG		*p* between Groups
	Pre-	Post-	Pre-	Post-	
**WBAH (Kg)**	40.2 (6.5)	37.4 (5.5) *	49.1 (8.5)	47.4 (8.3) *	0.151
**WBAS (Kg)**	32.5 (5.7)	35.4 (5.4) *	37.1 (6.8)	39.8 (8.0) *	0.985
**ΔWBA (Kg)**	7.7 (7.6)	2.0 (5.3) *	12.1 (9.2)	7.62 (11.2) *	**0.024**
**6MWT (m)**	194.3 (82.6)	308.4 (119.3) *	187.5 (84.2)	310.2 (105.1) *	0.897
**NRS (score)**	3.7 (2.1)	0.7 (0.3) *	3.19 (2.29)	1.1 (0.5) *	0.877

Data are reported as mean and (standard deviation) * significative comparison within group.

**Table 4:** Weight and WB sum in the groups.

	Pre			Post		
Group	Weight (kg)	Sum of WB on the Two Side (kg)	Δ of Weight and Sum of WB	Weight (kg)	Sum of WB on the Two Side (kg)	Δ of Weight and Sum of WB
**EC**	72.74	73	−0.26	70.74	72.8	−2.06
**CG**	86.29	86.2	0.09	84.29	87.2	−2.91

Data are reported as mean.

## Data Availability

The data presented in this study are available on request from the corresponding author. The data are not publicly available due to privacy reasons.

## Supplementary Materials

The following are available online at https://www.mdpi.com/1660-4601/18/7/3346/s1, File S1: Consort 2010 checklist.
